# Bridging the digital health divide: a narrative review of the causes, implications, and solutions for digital health inequalities

**DOI:** 10.1080/21642850.2025.2493139

**Published:** 2025-04-23

**Authors:** Max J. Western, Eline S. Smit, Thomas Gültzow, Efrat Neter, Falko F. Sniehotta, Olivia S. Malkowski, Charlene Wright, Heide Busse, Carmen Peuters, Lucia Rehackova, Angelo Gabriel Oteșanu, Ben Ainsworth, Christopher M. Jones, Michael Kilb, Angela M. Rodrigues, Olga Perski, Alison Wright, Laura König

**Affiliations:** a Centre for Motivation and Behaviour Change, University of Bath, Bath, UK; b University of Amsterdam/ASCoR, Amsterdam, The Netherlands; c Department of Work & Social Psychology, Maastricht University, Maastricht, The Netherlands; d Department of Theory, Methods & Statistics, Open University of the Netherlands, Heerlen, The Netherlands; e Department of Behavorial Sciences, Ruppin Academic Center, Emeq Hefer, Israel; f Public Health, Social and Preventive Medicine, Centre for Preventive Medicine and Digital Health (CPD), Medical Faculty Mannheim, University of Heidelberg, Mannheim, Germany; g School of Applied Psychology, Griffith University, Mount Gravatt, Australia; h Institute for Health Transformation, School of Nursing and Midwifery, Faculty of Health, Deakin University, Victoria, Australia; i Leibniz Institute for Prevention Research and Epidemiology – BIPS, Bremen, Germany; j Barcelona Institute for Global Health, Barcelona, Spain; k Department of Nursing, Midwifery, and Health, Northumbria University, Newcastle upon Tyne, UK; l Faculty of Psychology and Educational Sciences, University of Bucharest, Bucharest, Romania; m School of Psychology, Faculty of Environmental and Life Sciences, University of Southampton, Southampton, UK; n Centre for Preventive Medicine and Digital Health (CPD), Medical Faculty Mannheim, University of Heidelberg, Mannheim, Germany; o Department of Child Nutrition, Max Rubner-Institut, Federal Research Institute of Nutrition and Food, Karlsruhe, Germany; p Department of Psychology, Northumbria University, Newcastle upon Tyne, UK; q Herbert Wertheim School of Public Health and Human Longevity Science, University of California, San Diego, CA, USA; r Faculty of Social Sciences, Tampere University, Tampere, Finland; s Institute of Pharmaceutical Sciences, King's College London, London, UK; t Faculty of Psychology, University of Vienna, Vienna, Austria; u Faculty of Life Sciences: Food, Nutrition and Health, University of Bayreuth, Kulmbach, Germany

**Keywords:** Digital divide, inequalities, socio-ecological model, eHealth

## Abstract

**Background:** Digital health interventions have the potential to improve health at a large scale globally by improving access to healthcare services and health-related information, but they tend to benefit more affluent and privileged groups more than those less privileged.

**Methods:** In this narrative review, we describe how this ‘digital health divide’ can manifest across three different levels reflecting inequalities in access, skills and benefits or outcomes (i.e. the first, second, and tertiary digital divide)*.* We also discuss four key causes of this digital divide: (i)) digital health literacy as a fundamental determinant; (ii) other personal, social, community, and societal level determinants; (iii) how technology and intervention development contribute to; and (iv) how current research practice exacerbates the digital health divide by developing a biased evidence base. Finally, we formulate implications for research, policy, and practice.

**Results:** Specific recommendations for research include to keep digital health interventions and measurement instruments up to date with fastpaced technological changes, and to involve diverse populations in digital intervention development and evaluation research. For policy and practice, examples of recommendations are to insist on inclusive and accessible design of health technology and to ensure support for digital health intervention enactment prioritises those most vulnerable to the digital divide.

**Conclusion:** We conclude by highlighting the importance of addressing the digital health divide to ensure that as digital technologies' inevitable presence grows, it does not leave those who could benefit most from innovative health technology behind.

## Introduction

Digital health (also referred to as eHealth and mHealth) interventions use digital technology such as computers, smartphones, or wearable sensors, to promote health and well-being, support treatment, or improve illness management (Fatehi et al., 2020). Digital health interventions have the potential to improve health globally by improving access to health-related information, services, and products (Gibbons, 2005). According to the World Health Organization (WHO), digital tools can accelerate progress towards reaching the Sustainable Development Goals of *Good health and well-being* and *Reduced inequalities* (World Health Organisation, 2021). The rise in internet access and ownership of digital technology worldwide (The World Bank, 2022) means that digital health interventions can reach large numbers of people at relatively low cost (Gentili et al., 2022).

It is therefore not surprising that digital health interventions have been developed for a wide range of health domains, including the management of mental health problems, chronic physical conditions (Hamine et al., 2015), and in the promotion of health-related behaviours including smoking cessation (Griffiths et al., 2018), diet (Chen et al., 2020), and physical activity (Stockwell et al., 2019). Interest in digital health interventions was accelerated during the Coronavirus disease 2019 (COVID-19) pandemic that highlighted the need for interventions that could be rapidly delivered, updated, and scaled remotely (Peek et al., 2020). Health services around the world are recognising the potential value of virtual wards that can deliver many aspects of care to people’s homes, bypassing the need for attendance at hospitals or general practices (NHS England, 2023a). Key benefits such as the speed at which health advice or behavioural support can be received at any time and place, the ability to personalise and tailor content, inclusion of gamification features and the flexibility to manage health and well-being needs in an autonomous way, make these tools an attractive prospect for healthcare providers and digital health users.

In some contexts, digital health interventions have been shown to decrease existing inequality for people with lower socioeconomic status, such as in smoking cessation (Brown et al., 2014). However, too often-digital interventions serve to benefit the more affluent and privileged users more than the less-privileged populations, which is commonly referred to as the ‘digital health divide’ (Cornejo Müller et al., 2020). For example, in their systematic review evaluating the relative benefit amongst participants of randomised controlled trials, Western et al. concluded that digital interventions promoting physical activity in adults are effective for people of high, but not of low, socioeconomic status (Western et al., 2021). Similarly, another review reported that older adults and people in lower-skilled jobs and/or living in rural areas might benefit less from mobile weight-loss interventions than their younger counterparts, who hold higher-skilled jobs, and live in urban areas (Szinay et al., 2023). Much research has demonstrated that less-privileged populations, e.g. people with lower levels of education, lower income, older age, non-white populations, or those from rural neighbourhoods are less likely to start or continue using digital interventions – while uptake and usage are considered prerequisites for effectiveness (Chae, 2018; Chesser et al., 2016; Rodgers et al., 2019). These results support concerns that digital health interventions are not currently contributing to the World Health Organization’s goal of *Improving Health for All* (Makri, 2019). Given the accelerated drive by healthcare systems towards the use of digital tools and interventions for delivering substantial parts of care, it is vital that researchers, policy makers and practitioners ensure this changing landscape is equitable and inclusive (Latulippe et al., 2017; Sanders & Scanlon, 2021).

The aim of this paper is to present a narrative critical review of the literature of the digital health divide drawing upon literature relating to its key causes and mechanisms, followed by recommendations for research, policy, and practice aimed at narrowing the digital health divide. We intend for it to function as a means to raise awareness of the digital health divide amongst researchers, technology developers, and clinical practitioners interested in digital health to inform more equitable future practice. This work was reviewed and deemed exempt from formal ethics review by the Biomedical Sciences Research Ethics Committee at University of Bath (approval number: 5603-5923).

## The three levels of the digital health divide

One might simply argue that the digital health divide could reflect the already well-established inequalities in health, such as the gap in the burden of disease and life expectancy between a number of socio-demographic indicators including wealth, education, and ethnicity (Avan et al., 2019; O'Neill et al., 2014). However, there is accumulating evidence that digitalization is adding a new layer to these disparities, and that the presence of a digital health divide may exacerbate existing health inequalities (Jahnel et al., 2022). Observers have argued that online activities reflect economic, social, and cultural relationships that exist offline (Warschauer & Matuchniak, 2010), while healthcare advancements including digital technologies, which are not immediately available to all, can increase health inequalities (Watts, 2020). Accordingly, differences in the uptake of, engagement with and effectiveness of digital health interventions according to social inequality indicators need to be understood across three underlying levels of digital health divides.

First, digital health promotion and care require access to digital technology. Access to the internet and relevant devices such as computers and smartphones is linked to socioeconomic status, although the gap has been closing in recent years, at least in high-income countries. Moreover, the use of the internet and digital devices is becoming increasingly common in low- and middle-income countries (LMIC), as well as among disadvantaged groups globally (McCool et al., 2022) further supporting the call to use digital technology for remote communities that may suffer from a geographic inequality in accessing care. However, affordability is still an issue among people in LMICs, and the digital infrastructure is not always as proficient in developing regions (Hui et al., 2022). Disparities in access and infrastructure to health technology are known as the *first digital health divide*.

Second, for digital health promotion and care to be beneficial, knowledge and skills pertaining to the use of and engagement with digital services, e.g. digital health literacy and the capabilities to set-up and navigate technologies and websites, is essential (Neter & Brainin, 2012). This relates to both knowing how to effectively find and interpret digital health information (e.g. on websites), as well as how to appraise and apply the accessed information, and having the skills to successfully generate information and interact via Web 2.0 technologies (e.g. social media, messaging services) (Van De Belt et al., 2010). Disparities in the skill set required to navigate and interact with health technology form the *second digital health divide*.

Third, even in circumstances where barriers to access and interaction with health technology are removed and patients can make sense of the information and advice obtained from digital tools, not everyone will receive the same benefit in terms of health outcomes, e.g. improved health behaviours, reduced illness, or better management of chronic disease (Neter et al., 2018). Indeed, several studies have observed that socioeconomic status is a predictor of benefit from digital tools and interventions (Kontos et al., 2014; Western et al., 2021). Disparities in outcomes from digital technologies are referred to as the *third digital health divide*.

In the following, we provide a narrative review of the causes and mechanisms underpinning the digital health divide and discuss implications for policy and practice. We thereby summarise an expert discussion held during the 2022 annual conference of the European Health Psychology Society, which all authors attended. The primary ‘access’ digital health divide tends to be explained by digital infrastructural and economic factors and is a fundamental prerequisite for engagement, use and benefit. Therefore, we predominately focus our discussion on understanding the second and third digital health divide, which share multifaceted and more complex causal mechanisms that exist despite technological availability.

## Causes and mechanisms underpinning the digital health divide

**Figure 1.**
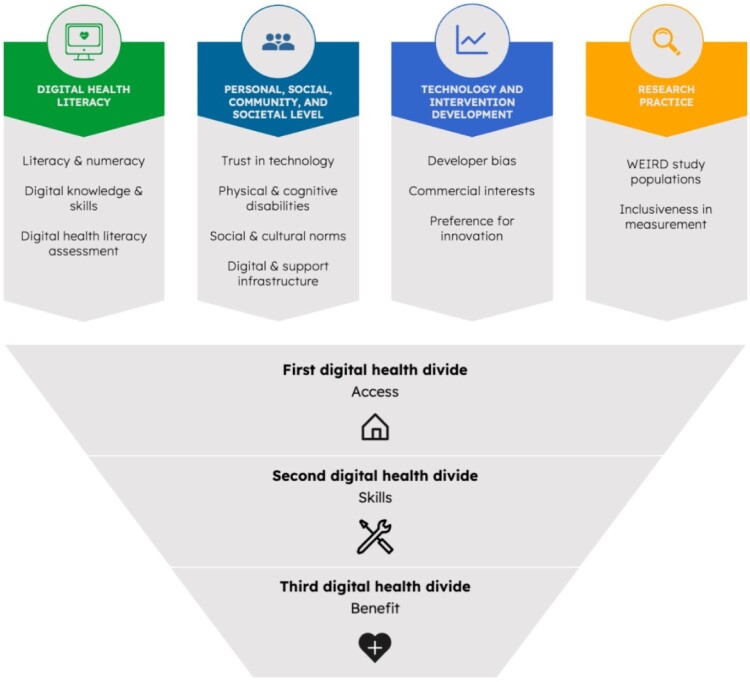
Causes underpinning the three levels of the digital health divide. Graphic with four columns in the top half summarising key causes of the digital health divide including (1) digital health literacy; (2) personal, social community, and societal; (3) Technology and intervention development; and (4) Research practice. The bottom half uses an upside down pyramid with three rows depicting the levels of digital divide: access, skills, and benefit.

Figure 1 provides an overview of some of the key factors that underpin the digital health divide, ranging from intrapersonal, to social and community factors, and research and design.

### Digital health literacy as a fundamental determinant

A central cause of health inequality is the levels of literacy and numeracy; skills closely aligned to socioeconomic variables including educational attainment, wealth, and migration (Saha, 2006; Ward et al., 2018). Health literacy, defined as the degree to which individuals can find, understand, and use information and services to make informed health-related decisions, and health numeracy, which relates to mathematical skills required to interpret health data, have been associated with numerous health outcomes (Berkman et al., 2011). Health literacy and numeracy predict engagement with digital resources for health-seeking behaviour (Jensen et al., 2010; Magsamen-Conrad et al., 2020). Online health information is, however, traditionally not optimally designed for people with low health literacy, who might need more cognitive resources and guidance to find, identify, and process the information. This may result in less recall of information and less desired attitudinal changes (Meppelink et al., 2016; Meppelink et al., 2017).

The concept of digital health (or eHealth) literacy was introduced to describe the ability to find, understand, and appraise health information from electronic resources and apply this knowledge to addressing or solving a health problem (Norman & Skinner, 2006a). Digital health literacy combines a broad set of knowledge and skills such as the capability to navigate digital interfaces that are more frequently present in affluent, well-educated populations than in more disadvantaged groups, which adds support to the argument that digital technology amplifies existing health inequalities (De Santis et al., 2021). A systematic review on 48 eHealth interventions (across 51 included studies) targeted at socially disadvantaged groups noted that eHealth literacy was generally overlooked in the development of such interventions (Cheng et al., 2020).

To identify those in need of intervention or support for improving digital health literacy, we need suitable measures. An issue with current self-report digital literacy scales, however, is that they fail to keep up with the fast-paced developments in digital technologies. For example, the measure most well-validated and frequently utilised to assess digital health literacy, the eHealth Literacy Scale (eHEALS) (Norman & Skinner, 2006b), was developed before the rise of social media, mobile devices, and smartwatches and does not reflect skills required for dealing with these aspects of digital health. Consequently, it is important to continue refining our measurement instruments in this sphere, as the ‘Health Literacy Scale – dealing with DIGital health Information’ (HLS_19_-DIGI) attempts (Lee et al., 2021; The HLS19 Consortium of the WHO Action Network M-POHL, 2022).

### Personal, social, community, and societal level determinants

As is the case for general health and health behavioural outcomes, the digital health divide is likely determined not only by digital health literacy but also by various other interacting factors, as outlined by the ‘Digital Rainbow’ model that explores individual lifestyle factors, social and community networks, and the wider cultural and environmental conditions (Jahnel et al., 2022; Lyles et al., 2023). At an individual level, attitudes towards technology, particularly trust in technology and a belief in the benefits that health technologies can offer, both contributing to the likelihood of adoption and engagement with digital health interventions, can differ according to age, ethnicity, and socioeconomic status (Pampel et al., 2010). In many cases, these factors have also been found to co-occur, particularly in people considered ‘digital immigrants’ rather than ‘digital natives’ (Kesharwani, 2020). To illustrate, mobile users who tend to rely more on themselves than on others when it comes to health-related decision-making have been found to be more eHealth literate, yet at the same time to express more privacy concerns when it comes to mobile health usage (Smit & Bol, 2020).

Indeed, a lack of trust creates a barrier to adoption and engagement with digital health tools, especially for older people, people of minoritized ethnic backgrounds, and those with lower education attainment (Fox & Connolly, 2018). Mistrust in technology tends to focus on access to personal and sensitive data and their (mis)use by third parties (Adjekum et al., 2018). Studies have also shown that people of lower socioeconomic status are relatively more vulnerable to reinforcement of poorer health behaviours by digital marketing algorithms, which may further lower their trust in (algorithm-driven) technology (Diviani et al., 2016; Panch et al., 2019). It is the responsibility of health technology creators to open the black box regarding data use and confidentiality, credibility of information sources, and the use of artificial intelligence in their applications.

Physical health can also play a role, where certain physical and cognitive disabilities can preclude an individual’s use of digital health tools. For example, age-related sensory (e.g. impaired vision and hearing) and cognitive decline (e.g. reduced processing speed) might influence older people’s needs regarding the mode in which information is presented (e.g. via textual, visual, or audio-visual modes) (Mason et al., 2021). It is important to note that older adults’ willingness and motivation to adopt digital technology and partake in a diverse range of online activities is improving. Ensuring that their needs pertaining to physical and cognitive decline are met is essential for enabling their increased participation in digital health (Quan-Haase et al., 2018; Tsai et al., 2015; van Boekel et al., 2017).

At a social and community level, the influence of family, friends, and peers on an individual’s health-related behaviour is recognized, including factors such as social and cultural norms towards (health) technology usage, social support for using technology – which can be both emotional and instrumental – and social modelling of technology use among relevant actors in the social environment (Konig et al. 2021; Hayat et al., 2017). To illustrate the interplay between socio-ecological levels, research has shown that supportive injunctive social norms, namely the perceived ‘rules’ that typify likeminded people’s behaviour or behaviours that are expected from others, can influence trust, perceived usefulness and intentions to adopt health technologies (Beldad & Hegner, 2018; Geber & Friemel, 2022; Yeoh et al., 2022). Accordingly, lower visible use and engagement in digital technology on behalf of the less privileged is unlikely to foster reaffirming injunctive and descriptive norms towards their uptake and engagement in digital health amongst these populations.

At the wider cultural and environmental level, important factors to consider are engagement with health professionals to enable patients to develop the capabilities to interact with technologies, and the often-digital channels through which digital health resources are advertised, which can alienate certain demographics who are not routinely exposed to digital advertising (Lyles et al., 2023). Similarly, local access and the level of investment in digital infrastructure contribute to the digital health divide, as are any discrepancies in the use of technology in other, non-health contexts, such as at work or in commerce (Koh et al., 2021). Policies on national digital infrastructure (e.g. free Wi-Fi for all), health service budgets, and resources for investing and supporting digital health roll-out can help mitigate digital inequalities. The implementation of cost-effective digital health interventions through healthcare systems is often suboptimal (Smit et al., 2015), and policies such as subsidised access and digital training are recommended to improve inclusivity.

### Technology and intervention development

To fully understand the causes of the digital health divide, and in turn identify the responsible stakeholders for combating it, it is important that we do not exclusively focus attention upon the characteristics of the disadvantaged individuals but also consider the contributing role of how technology and digital interventions are developed. It is not surprising, nor inconsequential, that most health technology is developed in affluent areas by academics, commercial technology developers, and otherwise highly educated people that likely share very similar backgrounds (Lyles et al., 2023). While academic and technical expertise is unquestionably essential for innovation and the progress towards more effective and affordable applications, the risk is inherent bias in the way these tools are designed, marketed, and supported that neglects the needs of the more disadvantaged people.

For example, if digital interventions focussed on reproduction-related decision-making are not developed for – let alone with – lesbian, gay, bisexual, transgender, queer/questioning, and intersex (LGBTQI+) populations, these groups will not be reached nor benefit, even if they have the necessary health literacy skills. Similarly, skin cancer detection app developers that do not include people of colour in the development process will most likely not reach this part of the target population (Whitehead et al., 2023). Furthermore, technology is – on the whole – developed to be profitable. Where insurance and welfare systems are not a viable source of financing, this implies that digital health interventions are mainly targeting large consumer bases with sufficient buying power. Traditionally, these are the people that are predominantly healthier, more educated, and have sufficient social capital – again leaving out the people that are less advantaged in these terms (Wang et al., 2021).

Other biases that the fast moving health technology landscape encounters include inherent assumptions that new technologies are better than old ones even if old technologies work well; optimism about technologies’ potential at the expense of their evidence base; instincts that more functionality, advanced features, and automation are better for users; and that technology is likely to be better or more efficient than humans (Greenhalgh, 2013). Perhaps one of the most important observations of Greenhalgh is that ‘the development and introduction [of health technologies] are occurring at a pace that far outstrips the evidence base for their efficacy and cost-effectiveness’ (Greenhalgh, 2013). Indeed, the haste at which digital health technologies are disseminated may also contribute to the digital health divide, since a speedy development process might limit opportunities for co-creation of digital health interventions with the intended target group, and especially the target groups that are traditionally underserved when it concerns health interventions or research.

### Research practice and the digital health divide

As a research community generating the evidence base for digital health technologies, it is important to be self-critical and describe how digital health research practice may contribute to the digital health divide. As with the development of technology described in the previous section, most digital health research has been conducted in (and by) WEIRD (i.e. Western, Educated, Industrialised, Rich and Democratic) populations – and even within WEIRD populations, mainly among the most privileged people (Szinay et al., 2023; Western et al., 2021). As this discussion highlights, however, it is not justified to assume that findings from research in WEIRD populations are generalizable across the board – especially as WEIRD populations only make up a small minority of the human population and most people are, in fact, not WEIRD (Henrich et al., 2010).

This resonates with evidence from scientific research in digital health that, for example, those with a higher socioeconomic status are easier to recruit to digital health interventions (Schneider et al., 2013), are less likely lost to follow-up during the course of a digital health intervention (Reinwand et al., 2015), and benefit more from interventions compared to lower socioeconomic participants (Western et al., 2021). For digital health researchers, it might be the path of least resistance to specifically focus research efforts on those populations that are more likely to provide the data necessary to evaluate their innovation. Yet, this may lead to biased study populations that show a high likelihood to engage with our interventions as intended and that will likely benefit most from them, thus optimising our digital health interventions specifically for these groups of people. It is probable that study populations are easiest to include in studies when they are most alike the researchers in terms of key characteristics. This may cause the digital health divide to go beyond WEIRD when it comes to the inclusiveness of study samples (e.g. people increasingly identify as non-binary) (Norori et al., 2021). This will further increase the existing inequity in the advantages that digital health might have to offer and widen the digital health divide.

Next to inclusiveness in the recruitment of study samples, inclusiveness in measurement also deserves attention. Especially when it comes to the measurement of psychological constructs, e.g. to measure digital health intervention effectiveness or working mechanisms, research traditionally relies on questionnaires, diaries, or other written records. The more fine-grained assessment of psychological outcomes is desired, the lengthier these questionnaires usually are, leading to a higher participant burden, which might in turn contribute to low levels of engagement and high attrition – both major challenges in digital health intervention research. As such, questionnaire length due to measurement comprehensiveness may disproportionately disadvantage participants who have more difficulty concentrating on or are less comfortable dealing with written questions (e.g. participants with attention deficit hyperactivity disorder or a specific learning disability such as dyslexia). Moreover, the fact that questionnaires usually include operationalisations that utilise natural language might be disadvantageous for linguistically diverse participants (e.g. people with a migration background or a lower educational level). For digital health research to be inclusive, solutions should be sought for such measurement issues.

## Implications

Based on our narrative synthesis of the causes and mechanisms underpinning the digital health divide, there are several implications for scientific research and policy and practice into this phenomenon. Table 1 provides a summary of the recommendations.

**Table 1.** Overview of recommendations for research, policy, and practice.

	Recommendations for research	Recommendations for policy & practice
1	Develop valid tools to identify users who are not likely to benefit from digital health interventions	Ensure optimal access to the Internet and digital devices at an international, national, and local levels
2	Develop and evaluate interventions that support vulnerable end-users and intermediaries in their use of digital health interventions	Insist on inclusive and accessible design of health technology
3	Co-create digital health interventions *with* vulnerable populations and intermediaries through participatory research methods	Ensure transparency and accessibility of data usage policies and information on effectiveness and potential unintended consequences of new technologies
4	Keep digital health interventions and measurement instruments up to date with fast-paced technological changes	Ensure support for digital health intervention enactment prioritises those most vulnerable to the digital divide
5	Recruit and engage diverse populations from within and beyond WEIRD populations in digital intervention development and evaluation research	Include evaluations of equality in decision-making around digital health intervention adoption and endorsement

### Implications for research

First, if we aspire to make digital health accessible and useful for all, it is important to investigate how to improve and/or accommodate low eHealth literacy skills, and to aid people with initial low levels of eHealth literacy to use digital health interventions to their benefit, now and in the future, since we do not want them to be left behind because of their initial disadvantage (Cheng et al., 2020). For instance, a meta-analysis of seven digital health literacy interventions amongst older adults found that they increased confidence and knowledge pertaining to eHealth, particularly if delivered face-to-face, theoretically grounded, and at least 4 weeks in duration (Dong et al., 2023). Yet, we emphasize the need for researchers to develop and evaluate theory-based interventions to provide all people who are vulnerable to the digital health divide, for example, those with a lower level of education, with the necessary skills and competencies to find, interpret, appraise and apply digital health information so they can benefit from digital interventions. As this may be challenging and not always possible given the resources available, it is additionally necessary to develop digital health interventions that are accessible for people with lower eHealth literacy skills, e.g. by adjusting the text density of the intervention and/or delivering (parts of) the intervention via more accessible modalities such as text messages, which do not require users to know how to use a smartphone or study app (Hall et al., 2015).

Second, to increase the adoption and engagement with technology, factors on different socio-ecological levels should be considered when developing, evaluating, and implementing digital health tools (McCall et al., 2022). For instance, next to increasing eHealth literacy, it is important to consider the role of trust, self-efficacy, motivation, and social influence and support for the use of digital health tools, not only at the level of the end-users but also at the level of the intermediaries working in healthcare systems (McCall et al., 2022). As such, we may also want to focus our research efforts on the development and evaluation of interventions that aim to support people in their use of the digital interventions meant to improve their health, e.g. by integrating the possibility to choose a delivery mode that fits their personal preferences best, which has been shown to increase users’ perceived active control, which in turn contributed to higher perceived relevance and website engagement, and reduced cognitive load, indirectly leading to a more positive website attitude and more information recall (Nguyen et al., 2020). Or, when it concerns the inclusion of digital health interventions in healthcare professionals’ repertoire, to create interventions that stimulate a positive social environment and increase compatibility of the digital health intervention with current practice (Leitlein et al., 2012). At the same time, the evaluation and selection of apps that can be used in healthcare, as well as technology set-up and troubleshooting with patients, and analysis and interpretation of data for use in clinical encounters, is a major challenge in light of the limited time available to healthcare providers outside of their clinical work (Wisniewski & Torous, 2020). To take on exactly these tasks, thereby helping both patient and provider, the idea to add an additional member to the care team, i.e. digital health navigator (Wisniewski & Torous, 2020), has been proposed.

Third, there is a strong need for co-creation in technology and intervention design, and to develop interventions *with* and not only *for* vulnerable populations. This recommendation is in line with recommendations articulated in models that aim to guide systematic intervention development (e.g. Intervention Mapping (Fernandez et al., 2019) and Design Thinking (Dragičević et al., 2023)). This may not necessarily be the quickest route to intervention development, since it may be challenging to enthuse end-users for participation in intervention development generally, but even more so for vulnerable end-users. Yet, this investment at the front end is highly likely to result in digital health interventions that match the needs and preferences of a diverse group of end-users much better, with more optimal health-related outcomes as a result. The establishment of a research panel consisting of people with a lower socioeconomic position might be one example of also reaching more vulnerable groups of people, thus aiding the achievement of this objective (NHS England, 2023b), see here for a successful example from the Netherlands (Nagelhout et al., 2023).

Fourth, both digital health interventions and digital health related measurement instruments should keep up to speed with the fast-paced advancement of digital technologies. As an example, as noted above, a new and better measurement instrument for eHealth literacy is needed, since the currently most widely used instrument has been found to not be up to date with the current digital context. This recommendation extends to other measurement instruments as well, especially when they aim to measure theoretical concepts that are specific to the fast-changing digital world, e.g. trust in (health) technology. For instance, whereas some attempts have been made to develop alternative proxies to lengthy, written questionnaires, novel more timely measurement instruments, the application of these new approaches does come with challenges (Short et al., 2022). For instance, while the use of routinely collected data may be helpful to identify people who could benefit from an intervention (e.g. those expressing a low mood on social media in case of a mental health intervention), the intellectual challenge lies in considering how to model these high-definition data to derive at meaningful summary statistics. Similarly, while the use of avatar selection may be an engaging way to examine user self-perceptions, a challenge relates to the determination of the extent to which avatar selection indeed resembles current or ideal self-perceptions (Short et al., 2022). Furthermore, future, inclusive measurement instruments need to minimise cognitive load and user burden, while ensuring that they do not themselves risk succumbing to the digital divide.

Finally, researchers should focus their explicit attention to recruiting and engaging diverse populations within and beyond WEIRD populations in their digital intervention development and evaluation research (König, Krukowski, et al., 2023). An example of how to increase awareness of this issue pertains to the use of the ‘Inclusivity & Diversity Add-On for Preregistration Forms’ when pre-registering digital health studies, as this may allow researchers to consciously review and share their considerations on socio-demographic characteristics in their study along its various stages, e.g. the team's composition, hypotheses, recruitment of participants, possibilities for data disaggregation, and even data sharing (Gültzow et al., 2023). Moreover, we should be aware that intersectionality, i.e. individuals with a combination of disadvantageous demographic factors related to for example age, gender, sexuality, and income, may make people especially vulnerable to the digital health divide. Research teams would also do well to consider solutions to common barriers to engagement across different sections of the research process, such as ensuring study adverts, procedures, travel, and intervention demands are accommodating of vulnerable sub populations (Krukowski et al., 2024).

### Implications for policy and practice

Importantly, narrowing the digital health divide requires input from health policy and decision makers as well as from stakeholders involved in commissioning, developing, and regulating digital health technologies. To tackle the primary digital health divide, initiatives to ensure optimal access to the Internet and digital devices is fundamental, considering inequalities at an international (e.g. health technology distribution in low- to middle-income countries), national (e.g. free/affordable Wi-Fi coverage and integration with affordable devices), and local level (e.g. reach of marketing strategies for digital health resources and interventions). Similarly, access to digital interventions can be facilitated at these levels. For instance, in Germany, the 2019 Digital Healthcare Act allowed doctors for the first time to prescribe digital interventions in the context of secondary and tertiary prevention. Costs for the interventions are then covered by health insurance, which also removes financial barriers (Gerke et al., 2020).

In terms of the second divide concerning knowledge and skills to appraise and use digital health technology, creators of digital health technologies should insist on inclusive and accessible design of health technology so that the language, content, and imagery does not alienate people that are more vulnerable, e.g. because of their older age, non-white backgrounds, neuro- or physical disability, or lower socioeconomic status. For digital health companies, especially given the fast-paced development of digital technology, systemic intervention development and evaluation may be too time-consuming in this regard, so more agile, iterative development methods such as the Multiphase Optimisation Strategy (Collins et al., 2007) might be preferred (König, Allmeta, et al., 2023). In addition, a less time-consuming alternative might be to establish a hub where digital health developers can access advice and support from behavioural sciences to make sure their final product ticks all of the boxes required for inclusive intervention design.

Consideration must also go to ensuring inclusivity at the different spheres of technological interaction, from the decision to adopt, to the interface and behaviours that the tool aims to support (Peters et al., 2018). Strategies to build trust in technology in populations who are less familiar or comfortable with it is essential to ensure wider engagement. Greater transparency and accessibility of data usage policies, as well as information on the effectiveness and potential unintended consequences of new technologies, would be useful ways to ensure inclusive standards are met. One study on the relationships between auditory and visual environmental factors in a video consultation setting and participants privacy concerns and willingness to disclose to the doctor (Bol et al., 2024), for example, showed that showed that the ability to see the doctor’s entire office led to lower information privacy concerns, which – in turn – were associated with increased willingness to disclose medical information, whereas wearing headphones by the doctor did not affect privacy concerns and self-disclosure tendency.

To tackle the third digital divide, relating to the (unequal) benefits people obtain from digital health interventions, one might consider the wider distribution of auxiliary resources that support or assist people in their offline health behaviours that the digital health interventions are designed to support. It is also important to recognize the vital role of social support for more vulnerable patient groups, and that ‘digital only’ interventions may not always be the most effective option for everyone (Courtois & Verdegem, 2014; Estrela et al., 2023). To this end, in might be necessary to take a more equitable approach that ‘makes use of’ the digital health divide by re-directing anyone that is affluent enough to use digital health to outside of healthcare, so that limited resources in healthcare can be spent on the less affluent people not able to use technology to its full advantage.

We also propose that frameworks guiding health technology assessment for informing the decisions of health systems to adopt or endorse digital tools for patients – or (un)healthy individuals more generally – to place much more emphasis on evaluations of equality and social perspectives (Kristensen, 2012). Similarly, regulatory frameworks must maintain pace with technological development, and consider acknowledging ‘exacerbation of digital inequalities’ as a safety issue that intervention developers are required to measure and mitigate.

## Conclusion

Due to the increasing digitisation of healthcare globally, moving parts of health promotion and care online seems to be inevitable. Digital delivery comes with a clear set of advantages for both healthcare providers and digital health users, e.g. the possibility to receive health advice or behavioural support at any time and place, the ability to personalise and tailor content and to include gamification features, as well as the flexibility to manage health and well-being needs in an autonomous way. Nevertheless, as the issue of the digital health divide highlights, digital solutions may not always be the most equitable option and effective for everyone. The mechanisms underpinning the digital divide are multifaceted and complex, with no simple solution. It is important to acknowledge that the digital health divide is not only a matter related to skills and motivation, and addressing structural issues such as access to devices, the evaluation mechanism of digital health interventions, and policies guiding their adoption are vital for a more equitable digital health landscape.

## Acknowledgements and author contributions

Similar to how digital health developers and behavioural scientists contributed to the digital health divide, the field might be very well suited to contribute to reducing this phenomenon. In an initial attempt to join forces in this respect, a group of behavioural scientists, health psychologists and other related experts came together during the 36th annual conference of the European Health Psychology Society (EHPS) conference, held in Bratislava, Slovakia, in August 2022. During a roundtable session, these scholars sought to raise awareness of social inequality in digital health promotion and stimulate further research and practical action. More specifically, based on group discussions, they aimed to (i) discuss how to better understand the reasons for the digital health divide and develop a research agenda that may also lead to practical solutions and (ii) acknowledge methodological challenges in studying the (reasons for) the digital health divide, and discuss potential ways to deal with these challenges. We used the results of this roundtable session as a basis for this paper and would therefore like to thank all participants for their valuable contributions. The first draft of the manuscript was produced by MJW, ESS, and LK. All named authors contributed to revising the manuscript and have approved the final version.
